# Social context, diversity and risk among women who inject drugs in Vietnam: descriptive findings from a cross-sectional survey

**DOI:** 10.1186/s12954-015-0067-9

**Published:** 2015-10-16

**Authors:** Oanh TH Khuat, Martha Morrow, Trang NN Nguyen, Gregory Armstrong

**Affiliations:** Center for Supporting Community Development Initiatives (SCDI), Hanoi, Vietnam; Centre for Mental Health, University of Melbourne, 3010 Victoria, Australia; Center for Promotion of Quality of Life (Life Center), Ho Chi Minh City, Vietnam

**Keywords:** PWID, Women, Vietnam, Gender, HIV, Risk, Cross-sectional survey

## Abstract

**Background:**

Women who inject drugs (WWID) are neglected globally in research and programming yet may be likelier than males to practise sexual and injecting risks and be infected with HIV and more stigmatised but seek fewer services. Little is known about characteristics, practices and nexus between drugs and sex work of WWID in Vietnam, where unsafe injecting has driven HIV transmission, and commercial sex and inconsistent condom use are prevalent. This was the first quantitative investigation of Vietnamese WWID recruited as injecting drug users. This article summarises descriptive findings.

**Findings:**

A cross-sectional survey was conducted among WWID in Hanoi (*n* = 203) and Ho Chi Minh City (HCMC) (*n* = 200) recruited using respondent-driven sampling. Characteristics varied within and between sites. Twenty-two percent in Hanoi and 47.5 % in HCMC had never sold sex. Almost all commenced with smoking heroin, some as children. Most injected frequently, usually alone, although 8 % (Hanoi) and 18 % (HCMC) shared equipment in the previous month. Some had sex—and sold it—as children; most had multiple partners. Condom use was high with clients but very low with intimate partners, often injecting drug users. HIV knowledge was uneven, and large minorities were not tested recently (or ever) for HIV. Nearly all perceived intense gender-related stigma, especially for drug use.

**Conclusion:**

This ground-breaking study challenges assumptions about characteristics and risks based on anecdotal evidence and studies among men. Most WWID were vulnerable to sexual HIV transmission from intimate partners. Interventions should incorporate broader sociocultural context to protect this highly stigmatised population.

## Findings

### Introduction

Vietnam reported in 1991 the first case of human immunodeficiency virus (HIV) infection; 25 years later, 227,114 people were living with HIV and 74,442 people had died of AIDS [1]. The epidemic has been slowed down in recent years from the annual new infections of over 30,000 in 2006–2007, but there were still 12,500 people newly diagnosed in 2013 [2].

The HIV epidemic in Vietnam was triggered and driven by drug injection. In the early 1990s, the annual proportion of newly diagnosed HIV cases among people who inject drugs (PWID) was as high as 87 % [3]. By the early 2000s, HIV prevalence among injecting drug users in Vietnam peaked at around 30 % before slowly and steadily reducing to around 10 % in 2014 as harm reduction was introduced and scaled up [4].

Women who inject drugs (WWID) tend to progress faster than males to dependence; inject more frequently; have intimate partners who inject, acquire and die from HIV/AIDS; and have greater combined risks, partly because many sell sex to purchase drugs [5–8]. Stigma may be greater than towards men who inject drugs (MWID) because ‘injecting drug use is often seen as contrary to the socially derived roles of women as mothers, partners and caretakers’ ([9], p. 19). Epidemic data shows that the share of drug injection as a mode of transmission has been reduced significantly from over 80 % in the 1990s to 35.4 % in the first 6 months of 2015 [4]. This indicates the ongoing significance of drug injection but also the increasing importance of sexual transmission. In such context, WWID as an HIV ‘bridge’ through cross-over of injecting and sex work (SW) is of epidemiological importance where commercial sex and inconsistent condom use are prevalent [10–15]. There is a dearth of research on WWID and of interventions that encompass drug use and wider health needs [7].

Little is known about characteristics, usage patterns, extent of sex work and HIV risks among WWID in Vietnam. Sentinel surveillance among PWID excludes females; most data on WWID is about SWs who inject [5, 12, 16, 17]. This paper reports descriptive findings from a cross-sectional survey, aimed to inform policy makers and programme managers about characteristics of WWID in the two major cities of Vietnam and their HIV-related behaviours so that policies and programmes can be adapted to produce stronger impacts on the HIV epidemic in Vietnam. The research, conducted in Hanoi and Ho Chi Minh city in 2010–2011, was funded through an Australian Development Research Award.

## Methods

An advisory group—consisted of representatives of WWID, HIV programme managers and public security officials—was set up to guide the study. Per advice of the group, participants were recruited from Hanoi and Ho Chi Minh city—the two largest cities with the highest numbers of people who inject drugs and also the highest concentration of WWID.

Women aged 18+ who injected at least once in the previous 6 months were recruited using respondent-driven sampling (RDS) [15, 17–19]. The sample size of 200/site was based on the assumed prevalence of 50 % for key responses (which would yield the biggest sample size), 95 % confidence interval, 8.5 % margin of error and design effect of 1.5. In each city, the recruitment started with nine ‘seeds’, balanced between age groups, HIV status and sex work involvement. Each participant was given three coupons to recruit others. Data collection was done at a drug user organisation’s office. Core members of the organisations provided information about the study; screened potential participants for eligibility, especially by checking injection marks and asked questions about injection practice; and monitored recruitment to avoid repeated participation. Interviewers were social researchers experienced in and comfortable with interacting with WWID. Participants got compensation of 150,000 Vietnam dongs (around 8 US dollars) for their contribution. In total, 203 WWID in Hanoi and 200 in Ho Chi Minh City (HCMC) participated.

Data were entered analysed by RDSAT v 6 [15] except constructing means (used SPSS v18). Approval was given by University of Melbourne’s Human Research Ethics Committee and the Hanoi investigator’s institutional review board.

## Results and discussion

### Characteristics

Mean age of WWID in Hanoi was 32.8 (18–54) years while in HCMC was 27.3 (18–35). Hanoian WWID on average had 7.9 (0–12) years of education and HCMCs had an average of 6.7 (0–13). SW was a main income source for almost two thirds in Hanoi but <30 % in HCMC. HCMC had more unemployed (Table 1). Nearly two thirds in Hanoi and 44 % in HCMC had ever married; similar proportions had children. Most did not live with a partner, and most partners used drugs. Most common accommodation in Hanoi was self-rented, and in HCMC was with family, but 10 % were homeless in HCMC (3 % in Hanoi).

**Table 1:** Sample characteristics

	Hanoi % (95 % CI) *n* = 203	HCMC % (95 % CI) *n* = 200
Main source of income		
Skilled worker	0.2 (0.0–0.7)	6.4 (0.0–8.0)
Non-agricultural labour (unskilled)	14.3 (6.9–23.6)	19.3 (13.8–25.5)
Salaried (clerical/sales/transport)	0.7 (0.0–1.6)	16.4 (9.7–26.9)
Petty business/trader/shop owner	7.6 (3.3–11.3)	1.7 (0.4–3.6)
Student	4.4 (0.0–6.3)	2.3 (0.9–4.2)
Sex work	62.7 (52.5–71.6)	29.5 (20.9–37.7)
Unemployed	8.0 (4.1–13.8)	33.0 (26.0–41.0)
Stealing	1.1 (0.0–1.6)	4.2 (2.2–6.5)
Living with regular partner	36.3 (26.1–46.4)	39.0 (31.9–45.6)
Partner uses drugs	65.2 (25.6–92.1)	75.9 (61.8–97.1)
Ever married	62.2 (53.9–72.4)	44.4 (36.2–52.7)
Have children	63.3 (54.7–72.3)	41.5 (32.6–50.1)
Accommodation this week		
Self-owned flat	5.8 (2.4–9.4)	5.5 (0.2–6.8)
Family-owned flat	24.1 (16.7–32.0)	46.7 (39.4–56.0)
Flat owned by partner	7.1 (2.5–11.5)	1.6 (0.4–3.3)
Self-rented place	56.1 (45.5–65.2)	35.0 (27.1–42.0)
Rented/owned by other	5.8 (2.1–11.2)	3.4 (1.4–5.7)
Hotel/guesthouse	0.2 (0.0–0.4)	1.6 (0.0–1.8)
On the street (homeless)	3.1 (0.0–4.4)	10.4 (6.8–18.3)

### Knowledge and testing

Knowledge about HIV transmission through tattoos and breastfeeding was inadequate, and one fifth in Hanoi and 40 % in HCMC believed they could identify an infected person by appearance. (Table 2) In HCMC, 29 % had not heard of sexually transmitted infections (STIs). Although viral hepatitis is a scourge among PWID [16], 82 % (Hanoi) and 70 % (HCMC) had never heard of Hep C, and <60 % knew of Hep B. Over one third knew nothing about HIV treatment. Among the 81 % (Hanoi) and 65 % (HCMC) ever tested for HIV, 35 % (Hanoi) and 40 % (HCMC) were untested for >1 year.

**Table 2:** HIV knowledge and testing

	Hanoi % (95 % CI)	HCMC % (95 % CI)
Ever heard of STIs	*n* = 201	*n* = 200
	93.6 (89.2–97.4)	71 (64.1–78.8)
Ways to prevent HIV (open question)	*n* = 195	*n* = 183
Avoid penetrative sexual intercourse	3.0 (0.7–5.0)	8.0 (3.5–13.4)
Always use a condom during vaginal sex	91.3 (86.6–97.0)	86.3 (79.3–92.6)
Always use a condom during anal sex	31.4 (22.2–40.2)	70.4 (63.4–78.2)
Avoid sharing injecting equipment	92.4 (88.1–96.2)	80.0 (72.7–87.3)
Avoid getting mosquito bites	0.6 (0.1–5.4)	8.8 (0.8–9.3)
Do not use shared clothes or eating utensils	2.3 (0.4–5.4)	2.3 (0.9–3.4)
Eat nutritious food	0.0 (-- --)	2.5 (0.0–2.8)
Have sex only with one uninfected partner	3.5 (1.1–5.7)	24.4 (18.5–30.8)
Avoid sharing needles to burn tattoos	11.2 (7.0–16.6)	13.6 (8.5–22.1)
Agrees HIV infection can be known by appearance alone	*n* = 200	*n* = 189
	20.9 (13.0–27.9)	39.9 (32.2–51.4)
Agrees treatment exists for HIV	*n* = 185	*n* = 200
	60.6 (51.5–71.3)	64.5 (56.2–71.4)
Agrees HIV+ woman can transmit virus to child via breastfeeding	*n* = 186	*n* = 200
	79.2 (44.0–15.4)	74.9 (67.4–81.6)
Have heard of hepatitis B	58.0 (51.3–67.5)	45.0 (36.3–53.0)
Have heard of hepatitis C	17.8 (12.4–25.0)	30.4 (24.0–37.7)
Ever had an HIV test	*n* = 201	*n* = 200
	81.3 (74.1–88.0)	64.8 (56.3–73.9)
When last took HIV test^a^		
Less than 1 year	64.6 (51.7–74.6)	59.9 (47.6–69.2)
More than 1 year	35.4 (25.7–47.8)	40.1 (31.0–52.9)

-- -- Unable to generate confidence interval in RDSAT

^a^Among those who have had an HIV test

### Gender and perceived stigma

Our sample perceived WWID (especially) and SWs as intensely stigmatised. The vast majority felt drug use or selling sex inhibited finding a non-injecting partner (Table 3).

**Table 3:** Perceived community attitudes towards injecting drugs and sex work

	Hanoi % (95 % CI)	HCMC % (95 % CI)
Agree society considers WWID to be ‘worse’ than MWID	*n* = 201	*n* = 200
	86.9 (77.7–91.4)	80.6 (74.4–86.5)
Perceived community views of WWID (>1 response permitted)	*n* = 203	*n* = 200
Bad character	88.3 (81.6–92.5)	89.2 (84.4–92.9)
Selfish	3.0 (1.0–5.3)	16.6 (11.3–24.7)
Irresponsible	12.8 (6.8–17.6)	15.4 (10.2–22.8)
Criminal	19.8 (14.2–26.5)	15.9 (10.3–20.7)
Feel afraid of them	51.1 (42.6–59.8)	43.0 (34.1–50.5)
Do not trust them	41.6 (31.6–49.7)	44.9 (38.2–54.7)
Assume they are sex workers	11.0 (6.7–15.7)	1.5 (0.2–3.5)
Feel sorry for them	4.3 (1.4–8.2)	1.0 (0.3–1.9)
Feel they are in a troubled situation	4.1 (0.8–6.4)	2.6 (0.0–6.7)
Perceived community views of SWs (>1 response permitted)	*n* = 203	*n* = 200
Bad character	88.3 (82.1–93.4)	62.7 (55.0–70.7)
Selfish	2.7 (0.8–5.0)	11.0 (6.0–17.0)
Irresponsible	11.6 (7.6–16.4)	13.6 (7.6–20.4)
Criminal	3.3 (-- --)	10.0 (5.3–15.5)
Feel afraid of them	19.9 (11.9–26.8)	22.5 (15.8–28.5)
Do not trust them	23.7 (14.9–30.5)	17.6 (11.5–22.9)
Feel sorry for them	17.9 (12.4–26.5)	25.7 (19.2–32.7)
Feel they are in a troubled situation	17.1 (11.7–22.2)	25.3 (18.8–33.1)
Perceived community views of female drug use versus sex work	*n* = 202	*n* = 200
Female drug use is worse than sex work	54.9 (47.7–67.0)	54.9 (47.5–63.9)
Sex work is worse than female drug use	11.5 (5.8–16.5)	11.6 (7.2–16.9)
Female drug use and sex work are equivalent	32.8 (23.9–41.6)	34.8 (26.1–44.5)
Agree it is more difficult for WWID to get a non-injecting partner	*n* = 199	*n* = 198
	93.7 (91.8–97.3)	83.2 (77.0–88.4)
Perceived difficulty for WWID to sell sex	*n* = 198	*n* = 196
Easier	1.1 (0.1–2.4)	22.1 (16.6–28.8)
Same	3.6 (0.9–4.4)	14.7 (9.2–20.7)
More difficult	93.5 (91.6–97.1)	61.2 (52.7–68.0)

-- -- Unable to generate confidence interval in RDSAT

### Injection and sexual risks

**Fig. 1:**
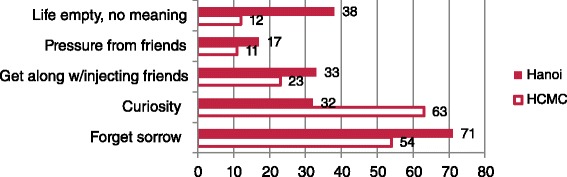
Reasons given (%) for starting to use drugs (>1 response acceptable)

Entry into drugs and sex was varied. More than 70 % of our sample cited friends, and less than one quarter cited husband/boyfriend, as those who introduced them to drugs (Table 4). Almost all started with heroin, mostly smoked/inhaled apart from 26.7 % (CI 18.6–35.5) in Hanoi and 13.5 % (CI 7.1–21.2) in HCMC who commenced with injecting. Similar reasons were offered but different proportions; for example, ‘forget sorrow’ was most common in Hanoi and ‘curiosity’ in the younger HCMC sample (Fig. 1). Mean age of first use was 24 (13–47) in Hanoi and 19.8 (11–33) in HCMC. Around 28 % in Hanoi and 62 % in HCMC used by age 20, and 0.5 % and 12 % were under 16, respectively; HCMC’s younger profile suggests initiation is starting earlier.

**Table 4:** Injecting behaviour by site

	Hanoi % (95 % CI)	HCMC % (95 % CI)
Who introduced you to drug use?	*n* = 203	*n* = 200
Friend	70.6 (62.3–78.8)	72.6 (66.0–79.0)
Boyfriend/husband	24.6 (17.1–31.5)	11.6 (7.0–17.0)
Drug dealer	0.0 (0.0–0.0)	0.6 (0.1–1.2)
Sibling	1.8 (0.0–4.5)	5.8 (2.7–9.4)
Client	1.5 (0.0–4.4)	1.4 (0.0–3.6)
Age first drug use	*n* = 203	*n* = 200
<16	0.5 (0.1–0.9)	12.1 (7.3–18.0)
16–20	27.8 (18.7–37.7)	49.7 (41.4–56.4)
21–25	40.3 (30.3–50.4)	29.4 (23.4–37.6)
26+	31.4 (22.7–40.7)	8.8 (4.4–12.8)
Two most common injecting locations	*n* = 203	*n* = 200
Own house	87.1 (82.0–93.5)	50.2 (41.0–58.1)
Public toilet	37.8 (29.0–49.6)	29.2 (21.7–36.3)
Street or park	18.4 (10.1–26.0)	37.6 (30.5–45.9)
Guesthouse or hotel	40.6 (33.4–50.6)	5.7 (2.9–9.1)
Home of male partner	20.1 (12.6–30.3)	4.5 (2.6–6.9)
Frequency of injecting (past month)	*n* = 203	*n* = 200
4–6 times a week	4.7 (0.0–6.6)	1.0 (0.0–1.6)
About once daily	8.8 (5.1–13.0)	18.3 (10.7–25.8)
2–3 times daily	60.7 (50.6–71.0)	59.2 (50.8–67.6)
4+ times daily	19.2 (12.3–27.6)	21.5 (15.4–28.5)
Shared needles and syringes (NS) in past month	*n* = 196	*n* = 199
	8.3 (1.8–14.8)	18.4 (12.9–24.7)
At last injection	*n* = 203	*n* = 198
No others present	68.0 (57.5–77.4)	61.4 (52.8–69.8)
One or more others present	32.0 (22.7–42.6)	38.6 (30.2–47.3)
Shared drugs^a^	34.9 (17.2–67.3)	39.1 (21.4–53.0)
Shared mixing water^a^	38.0 (7.9–72.7)	59.7 (43.6–88.0)
Shared NS occasion^a^	24.8 (2.9–50.2)	33.6 (15.8–50.8)

^a^Among those who did not inject alone

Around one fifth of our participants in both cities reported injecting at least four times a day: 19 % Hanoi, 21.5 % HCMC. Women primarily injected alone, most often in their homes (Hanoi 87 %, HCMC 50 %), guesthouses in Hanoi, streets/parks in HCMC and public toilets in both. Needle sharing in the previous month was 8.3 % in Hanoi and 18.4 % in HCMC. Reasons for sharing (>1 permitted) were craving, convenient and to express love/trust or share fate.

Mean age at first intercourse was 18.4 years (8–30) in Hanoi, 17.9 years (11–28) in HCMC, but some reportedly had sex as children (Table 5). In HCMC, 24 % had sex before age 16 (4 % in Hanoi).

**Table 5:** Sexual practices

	Hanoi % (95 % CI)	HCMC % (95 % CI)
Age at first sexual intercourse	*n* = 202	*n* = 181
Less than 16	4.2 (0.7–7.5)	24.4 (15.5–30.3)
16	15.4 (9.3–22.7)	19.9 (12.1–26.8)
17	14.1 (7.8–18.5)	13.7 (5.8–17.4)
18	26.2 (19.2–35.8)	12.2 (8.3–18.3)
19	11.6 (6.7–16.2)	13.1 (7.6–21.6)
20+	28.5 (21.8–38.5)	16.7 (13.4–27.8)
Ever sold sex	*n* = 202	*n* = 184
	77.6 (68.9–84.2)	52.5 (41.5–63.5)
Age first sold sex	*n* = 158	*n* = 102
16 or less	3.3 (-- --)	19.5 (8.4–28.0)
17–20	16.5 (6.6–21.6)	50.4 (31.8–59.3)
21–25	40.0 (28.8–59.1)	19.9 (12.0–36.4)
26–30	27.1 (18.1–40.7)	8.0 (4.5–17.6)
31+	13.1 (5.3–23.5)	2.3 (0.0–7.6)
Ever sold sex for partner’s drugs^a^	*n* = 158	*n* = 106
	39.6 (25.9–56.7)	25.3 (14.6–37.6)
Introduced to selling sex by …^a^	*n* = 158	*n* = 106
Own decision	36.2 (25.9–52.7)	38.4 (22.9–44.9)
Female who injected drugs	34.5 (23.4–49.0)	19.9 (13.3–34.2)
Female who did not inject drugs	33.6 (18.1–50.3)	4.7 (0.6–7.8)
Sexual partner or husband	1.6 (0.0–1.8)	21.6 (10.2–36.4)
Family member (except for husband/partner)	0.0 (0.0–0.0)	16.5 (0.0–17.0)
Main locations to find clients^a^	*n* = 158	*n* = 106
Bar/nightclub	18.6 (7.4–33.5)	9.5 (5.3–16.3)
Public place (other than highway)	39.6 (28.9–55.7)	44.5 (31.9–57.1)
Service bar	6.9 (2.4–12.0)	5.5 (0.5–6.0)
Brothel	1.4 (0.0–2.7)	6.6 (0.6–22.1)
Hotel/guesthouse	7.5 (3.2–13.3)	5.9 (2.2–12.6)
Highway/street	16.1 (4.5–31.1)	13.4 (7.2–23.6)
Home	5.1 (0.0–8.2)	7.6 (0.0–9.6)
Have a regular client^a^	*n* = 156	*n* = 95
	63.6 (44.9–72.6)	55.3 (42.0–65.8)
Used condom at last sex with husband/boyfriend	*n* = 176	*n* = 173
	17.5 (11.2–25.9)	32.3 (22.9–40.2)
Used condom at last sex with one-time client^a^	*n* = 156	*n* = 95
	82.5 (59.6–94.3)	91.9 (83.7–98.5)
Used condom at last sex with regular client^b^	*n* = 118	*n* = 56
	85.3 (60.4–97.9)	86.3 (53.2–92.2)

^a^Among those who had ever sold sex

^b^Among those who had regular clients

-- -- Unable to generate confidence interval in RDSAT

**Fig. 2:**
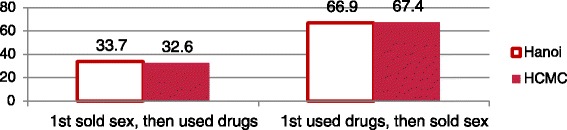
Sequence of using drugs, selling sex (among those who had sold sex) (%) (Hanoi *n* = 158, HCMC *n* = 106)

Over one fifth in Hanoi and nearly half in HCMC reported they had never sold sex. Among those who had, two thirds were using drugs before they first sold sex (Fig. 2).

Some sold sex as children. Mean age of first SW was 25 (14–52) in Hanoi and 20 (13–33) in HCMC, where almost 20 % sold sex before age 17 (3.3 % in Hanoi). Substantial minorities sold sex to buy drugs for partners. Over one third claimed SW was their decision. In Hanoi, 85 % (81.3–90.9), and HCMC, 72 % (63.3–79.6), had sex in the past month; mean number of partners was 44 (1–180) in Hanoi, 12 (1–100) in HCMC. Clients were found mainly in public places, rather than brothels or bars.

Condom use was high with clients. However, 76 % of sexually active women in Hanoi and 83 % in HCMC had ≥1 ‘husband/boyfriend’, many/most of whom used drugs; just 17.5 % in Hanoi and 32 % in HCMC used condoms the last time.

### Limitations

Reporting of certain behaviours may be influenced by recall and social desirability bias. RDS recruits through peer networks; hence, some types of WWID, e.g. those who rarely interact with others, may not be sampled. Fears of facing the police (for doing sex work or using drugs) might have prevented some WWID to participate. Also, lack of a known sampling frame precludes certainty about generalisability.

## Discussion

Participants’ demographic data reflects the diversity of WWID (age range, socio-economic status, living arrangement, etc.), and the North–South differences imply different strategies are needed to reach and to deliver interventions to them.

However, common issues (and needs) of WWID were identified through the study: being single mothers, had sex or sold sex as a child, heavily dependent on drugs with a high frequency of injection, not using condom with intimate partners–multiple of them–most/all injectors with high probability of having HIV, inadequate knowledge on HIV transmission, suboptimal access to HIV testing, lacking knowledge on STI and viral hepatitis and high perceived stigma from society. Programmes to prevent blood-borne infections should be intensified among WWID. Psychological support, counselling, family planning and parenting skills are among interventions needed to address their different immediate needs.

From these WWID, we learn that drug use led some of them to sex work. Drug-dependent treatment would be an important intervention strategy to prevent this. But we also learn that not all WWID sell sex, so programmes targeting sex workers would not reach many of the WWID.

Given the epidemiological context in Vietnam where injection still plays an important role while sexual transmission is gradually becoming the most important mode of transmission, intervention for bridging groups such as WWID should be prioritised if the HIV epidemic in Vietnam is to be stopped.

## Abbreviations

HCMC: Ho Chi Minh City
HIV: human immunodeficiency virus
MWID: men who inject drugs
NS: needles and syringes
PWID: people who inject drugs
RDS: respondent-driven sampling
STI: sexually transmitted infection
SW: sex work/er
WWID: women who inject drugs
